# Cost‐effectiveness of first‐line versus second‐line use of domestic anti‐PD‐1 antibody sintilimab in Chinese patients with advanced or metastatic squamous non‐small cell lung cancer

**DOI:** 10.1002/cam4.5440

**Published:** 2022-11-13

**Authors:** Rihua Cheng, Zhen Zhou, Qiao Liu

**Affiliations:** ^1^ Department of Pharmacy Brain Hospital of Hunan Province (The Second People's Hospital of Hunan Province) Changsha Hunan China; ^2^ Menzies Institute for Medical Research University of Tasmania Hobart Tasmania Australia; ^3^ Department of Pharmacy The Second Xiangya Hospital, Central South University Changsha Hunan China

**Keywords:** China, cost‐effectiveness, domestic anticancer drugs, NSCLC, sintilimab

## Abstract

**Background:**

Programmed cell death protein‐1/programmed cell death ligand‐1 (PD‐1/L1) inhibitor is a promising therapeutic option that can be used as either a first‐line or second‐line treatment for driver‐negative advanced or metastatic squamous non‐small cell lung cancers (sqNSCLC). However, reuse of PD‐1/L1 inhibitor in second‐line beyond progression after the first‐line is generally not recommended. Therefore, oncologists face challenges in making a proper decision of using PD‐1/L1 inhibitor. This analysis aimed to determine whether it is more cost‐effective to use sintilimab, a domestic anti‐PD‐1 drug in China, as a first‐line treatment than reserving it until second‐line.

**Methods:**

We conducted a cost‐effectiveness analysis to compare the use of sintilimab in the first‐line setting with reserving its use until the second‐line for driver‐negative advanced or metastatic sqNSCLC from the perspective of the Chinese healthcare system. A Markov model composed of five main mutually independent health states and three temporary health states was established to simulate patients' clinical trajectory. Transition probabilities, including disease progression, survival, and adverse events‐related treatment discontinuation, were estimated using data from the ORIENT‐12, ORIENT‐3, and ALTER0303 clinical trials. The robustness of the model was assessed using deterministic sensitivity analysis (DSA) and probabilistic sensitivity analyses.

**Results:**

Reserving the use of sintilimab until the second‐line was associated with a greater effectiveness (1.52 vs. 1.37 quality‐adjusted life‐years [QALYs]) and a higher healthcare cost ($12,203 vs. $14,045) compared with the first‐line sintilimab, resulting in an incremental cost‐effectiveness ratio (ICER) of $12,693 per QALY. The results of DSA suggested that variations in all parameters did not result in the ICERs surpassing the willingness‐to‐pay threshold of $35,663/QALY.

**Conclusions:**

For Chinese patients with driver‐negative advanced or metastatic sqNSCLC, reserving the use of sintilimab until the second‐line represents a cost‐effective treatment strategy compared with the first‐line treatment. This finding is useful to inform Chinese healthcare policymakers regarding the optimized treatment strategies of use of domestic PD‐1/L1 inhibitors sintilimab.

## Graphical abstract

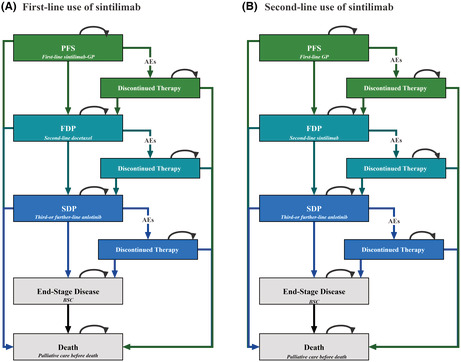

## 1 INTRODUCTION

Lung cancer is one of the major cancer types that leads to a high mortality rate globally. China has a greater number of lung cancer patients compared with other countries and this number is continuing to grow. The new cases in China was estimated to be 816,000 in 2020.^1^, ^2^ Squamous non‐small cell lung cancers (sqNSCLCs) account for about one‐quarter of all lung cancers,^3^ of which the majority cases are in an advanced stage or present with metastases when being diagnosed.^4^ Most sqNSCLCs do not have genetic aberrations, which makes the patients not be able to benefit from promising targeted therapies.^5^ Programmed cell death protein‐1/programmed cell death ligand‐1 (PD‐1/L1) inhibitor immunotherapies have shown a remarkable performance in treating driver‐negative advanced or metastatic sqNSCLC and they have now become the mainstay therapies in this patient group.^6^

Between 2018 and 2019, the Chinese National Medical Products Administration successively approved three imported anti‐PD‐1/L1 drugs (pembrolizumab, atezolizumab and nivolumab) for advanced or metastatic sqNSCLC.^7^ Despite the compelling clinical evidences showing great efficacy of these imported anticancer drugs, the prohibitively high costs make these drugs unaffordable for the majority of patients.^8^ As a consequence, there is an urgent need for more affordable anti‐cancer treatments with a comparable clinical efficacy to meet the unmet medical need of Chinese patients.^9^ Camrelizumab, tislelizumab, and sintilimab are novel PD‐1/L1 inhibitors that were independently developed by China and has been marketed successively.^10^, ^11^, ^12^ Although anti‐PD‐1/L1 therapies have been widely used in the first‐ and second‐line settings for patients with advanced or metastatic sqNSCLC, they are generally not recommended for a reuse in the second‐line treatment beyond progression after the first‐line.^13^ There is a clinical uncertainty regarding whether PD‐1/L1 inhibitors should be used as a first‐line treatment or reserved until the second‐line to maximize its clinical benefits.

Sintilimab is the first domestic anti‐PD‐1 drug proving its clinical efficacy in both first‐ and second‐line settings for advanced or metastatic sqNSCLC. The ORIENT‐12 clinical trial (ClinicalTrials.gov Identifier: NCT03629925) investigated the effect of adding sintilimab to gemcitabine/platinum in patients with advanced or metastatic sqNSCLC, and reported an improved progression‐free survival (PFS) and a favorable safety profile for sintilimab‐containing regimen.^14^ More recently, the ORIENT‐3 study (ClinicalTrials.gov Identifier: NCT03150875) assessing the efficacy and safety of sintilimab versus docetaxel in the same patient population after the first‐line platinum‐based therapy failed to achieve target goal, similarly showed a favorable net clinical benefit of sintilimab.^15^ Based on these results, sintilimab has become a key treatment of the stand‐of‐care for driver‐negative advanced or metastatic sqNSCLC.^15^

To explore the appropriate timing of using sintilimab, we conducted a cost‐effectiveness analysis to compare the use of sintilimab in the first‐line setting with its use until the second‐line for driver‐negative advanced or metastatic sqNSCLC from the perspective of the Chinese healthcare system.

## 2 METHODS

### 2.1 Overview

We performed this cost‐effectiveness analysis using a Markov model. Markov modeling is a statistical method that simulates the clinical trajectory of a hypothetical patient cohort, and then projects the cumulative clinical and economic outcomes within a fixed time horizon through a set of model inputs. This analysis used published data to inform the model and thus does not require the ethic approval from Chinese ethics review committee. Our study followed the China Guidelines for Pharmacoeconomic Evaluation.^16^

### 2.2 Model construction

**FIGURE 1:**
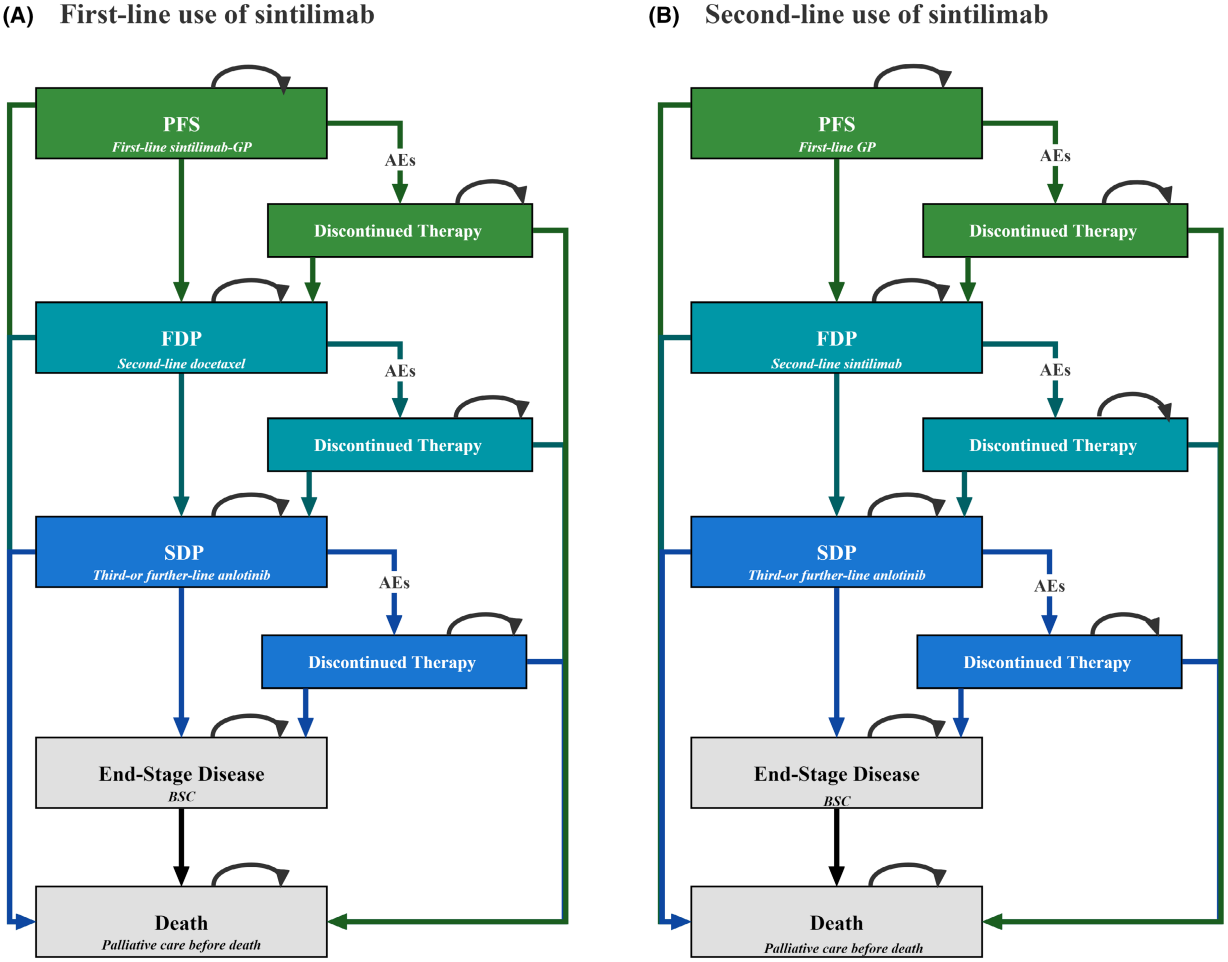
Diagram of Markov model. AEs, adverse events; BSC, best supportive care; FDP, first disease progression; PFS, progression‐free survival; SDP, second disease progression.

As illustrated by the transition state diagram in Figure 1, the Markov model consisted of five main mutually independent health states: PFS, first disease progression, second disease progression, end‐stage disease, and death. A hypothetical cohort of patients with driver‐negative advanced or metastatic sqNSCLC was created to mirror the subjects recruited in the ORIENT‐12 trial.^14^ The Markov cycle length was set to 3 weeks to reflect the real‐world administration interval, and the time horizon was set to 20 years to ensure that the model still runs after more than 99% of participants in the cohort reached the death outcome.

All patients started from the PFS health state and were randomly assigned to first‐line sintilimab plus gemcitabine/platinum (sintilimab‐GP) or GP alone. Individuals who progressed during the use of first‐line sintilimab‐GP were proceeded to second‐line docetaxel, and those who progressed during the use of first‐line GP were proceeded to second‐line sintilimab.^15^

Third‐or further‐line therapy was made identical in both groups and were modeled as anlotinib treatment.^13^, ^17^ Considering that the treatment discontinuation might occur due to intolerable drug toxicity before the disease progresses,^14^, ^15^, ^17^ three temporary health states named “discontinued therapy” were established to reflect clinical practice. Individuals whose cancers progressed on anlotinib treatment would enter an end‐stage disease health state and receive the best supportive care (BSC). Patients were recommended for palliative care when approaching death.^13^ Treatment dosage and administration schedule for each line treatment are detailed in Table S1.

### 2.3 Transition probabilities

Transition probabilities between Markov health states were estimated using published model‐fitting technique.^18^, ^19^ Using the method described in the study by Guyot et al.^20^ Kaplan–Meier (KM) curves extracted from the clinical trials were digitized by the GetData Graph Digitizer software (version 2.26; http://www.getdata‐graphdigitizer.com/index.php) and were then used to reconstruct patient‐level data. Subsequently, the optimal parameter model used to fit these reconstructed data was chosen among the exponential, Weibull, log‐logistic, lognormal and Gompertz distributions based on the statistical measures of goodness‐of‐fit analysis. Details regarding the model fit are presented in Figures S1–S3 and Table S2. The computed model parameters were then used to estimate transition probabilities between the five main Markov health states. The transition probabilities for temporary health states were derived from data on treatment discontinuation related to adverse events (AEs) (Table S3). Model inputs for transition probabilities estimations are summarized in Table 1.

### 2.4 Costs

The direct medical costs were covered, including drugs acquisition costs, AEs management costs and general cancer management costs. Drug acquisition costs were estimated based on the latest bid‐winning price, which is available from a public national database (https://www.yaozh.com/).^21^ The treatment dosage was determined according to patients' physical characteristics. In our analysis, we used a mean body surface area of 1.72 m^2^ and a mean creatinine clearance rate of 70 ml/min for model patients.^19^, ^22^ In this model, all grade III/IV AEs associated with first‐line treatments were considered. To calculate AE management cost for first‐line treatments, we first multiplied the frequency of each AE reported in the clinical trials by the corresponding treatment cost derived from local comprehensive hospitals, and then added all the products to get a sum. Details concerning the calculation of III/IV AEs management costs are provided in Table S4. The general cancer management costs, consisting of routine follow‐up cost, BSC cost and palliative care cost, were taken from previous literature.^17^ All cost inputs and the corresponding sources are detailed in Table 1.

### 2.5 Health state utility

Treatment effectiveness was measured by quality‐adjusted life‐years (QALYs), which is a discounted sum of Chinese‐specific utilities assigned to each health state.^23^ Experiencing toxicity during the first‐line treatment was found to be associated with disutility and was therefore considered in the model.^24^ Similar to the AEs management costs, we calculated disutility for first‐line treatments as a frequency‐weighted aggregate based on the utility decrements reported for these corresponding AEs (Table S4).

### 2.6 Statistical analysis

The model was created and analyzed using TreeAge Pro Healthcare software (version 2021, https://www.treeage.com/) and R software (version 4.0.4, http://www.r‐project.org). The relative cost‐effectiveness between the two competing strategies was measured using an incremental cost‐effectiveness ratio (ICER), which was calculated as the incremental healthcare costs consumed by each additional QALY. We reported the cost in 2021 USD (1 USD was equivalent to 6.4512 CNY) and discounted both the costs and QALYs at an annual rate of 5%.^16^ We set a willingness‐to‐pay (WTP) threshold as three times of China's per capita GDP in 2021 ($35,663 per QALY).^8^, ^16^ An ICER lower than the WTP threshold was considered cost‐effective.

To identify the most influential parameter, deterministic sensitivity analysis (DSA) was performed to investigate the sensitivity of our model results to the variations in input parameters. Each parameter was varied between the upper and lower limits listed in Table 1. Health state utilities were varied within their corresponding lower and upper 95% confidence intervals (CIs); costs, AEs‐related transition probabilities and disutilities, and patient's physical characteristics parameters were varied within plus or minus 50% of the baseline values; discount rate was varied from 0% to 8%.^16^ To verify the robustness of the model, probabilistic sensitivity analyses (PSA) were performed by simultaneously sampling parameters from the appropriate distributions recommended by the ISPOR‐SMDM Modeling Good Research Practices Task Force.^25^ During PSA, 1000 Monte Carlo simulations were employed to compute 1000 cost and QALY estimates for each strategy.

**TABLE 1:** Model inputs

Variable	Baseline value	Range	Distribution	Source
Survival				
OS for first‐line sintilimab‐GP	Log‐logistic *θ* = 0.001495; *κ* = 1.977611	Fixed in DSA	Fixed in PSA	Estimated^a^
OS for first‐line GP	Log‐logistic *θ* = 0.012050; *κ* = 1.388800	Fixed in DSA	Fixed in PSA	Estimated^a^
OS for second‐line sintilimab	Log‐logistic *θ* = 0.009967; *κ* = 1.617333	Fixed in DSA	Fixed in PSA	Estimated^a^
OS for second‐line docetaxel	Log‐logistic *θ* = 0.022400; *κ* = 1.520600	Fixed in DSA	Fixed in PSA	Estimated^a^
OS for third‐ or further anlotinib	Weibull *λ* = 0.043830; *γ* = 1.041130	Fixed in DSA	Fixed in PSA	Estimated^a^
PFS for first‐line sintilimab‐GP	Log‐logistic *θ* = 0.009008; *κ* = 2.158257	Fixed in DSA	Fixed in PSA	Estimated^a^
PFS for first‐line GP	Log‐logistic *θ* = 0.000821; *κ* = 3.689725	Fixed in DSA	Fixed in PSA	Estimated^a^
PFS for second‐line sintilimab	Log‐logistic *θ* = 0.109400; *κ* = 1.197400	Fixed in DSA	Fixed in PSA	Estimated^a^
PFS for second‐line docetaxel	Log‐logistic *θ* = 0.090110; *κ* = 1.843920	Fixed in DSA	Fixed in PSA	Estimated^a^
PFS for third‐ or further anlotinib	Weibull *λ* = 0.062080; *γ* = 1.228680	Fixed in DSA	Fixed in PSA	Estimated^a^
1‐Cycle probability of treatment discontinuation due to AEs				
First‐line sintilimab‐GP	0.003701	0.001850–0.005551	Beta	Estimated^b^
First‐line GP	0.003472	0.001736–0.005207	Beta	Estimated^b^
Second‐line sintilimab	0.007897	0.003948–0.011845	Beta	Estimated^b^
Second‐line docetaxel	0.004685	0.002343–0.007028	Beta	Estimated^b^
Third‐ or further anlotinib	0.007211	0.003606–0.010817	Beta	Estimated^b^
Costs (US$)				
Sintilimab per 200 mg	334.82	167.41–502.23	Gamma	Local charge
Gemcitabine per 1.0 g/m^2^	70.53	35.26–105.79	Gamma	Local charge
Cisplatin per 75 mg/m^2^	8.80	4.40–13.20	Gamma	Local charge
Carboplatin per 5.0 mg/ml/min	1.30	0.65–1.95	Gamma	Local charge
Docetaxel per 75 mg/m^2^	39.53	19.76–59.29	Gamma	Local charge
Anlotinib per 168 mg	665.97	332.99–998.96	Gamma	Local charge
Routine follow‐up per cycle	55.60	27.80–83.40	Gamma	^19^
BSC per cycle	337.50	168.75–506.25	Gamma	^19^
Palliative care per cycle	2627.80	1313.90–3941.70	Gamma	^19^
AEs management cost for first‐line sintilimab‐GP	2564.35	1282.18–3846.53	Gamma	Estimated^c^
AEs management cost for first‐line GP	2412.56	1206.28–3618.84	Gamma	Estimated^c^
Utilities				
PFS health state	0.856	0.718–0.994	Beta	^23^
FDP health state	0.768	0.595–0.941	Beta	^23^
SDP health state	0.703	0.545–0.861	Beta	^23^
End‐stage disease health state	0.703	0.545–0.861	Beta	^23^
AEs disutility for first‐line sintilimab‐GP	0.100	0.050–0.150	Beta	Estimated^c^
AEs disutility in first‐line GP	0.097	0.049–0.146	Beta	Estimated^c^
Other				
Discount rate (%)	5	0–8	Fixed in PSA	^16^
Body surface area (m^2^)	1.72	0.86–2.58	Normal	^19^
Creatinine clearance rate (ml/min)	70	35–105	Normal	^22^

Abbreviations: AEs, adverse events; BSC, best supportive care; DSA, deterministic sensitivity analysis; FDP, first disease progression; GP, gemcitabine plus platinum; OS, overall survival; PFS, progression‐free survival; PSA, probabilistic sensitivity analyses; SDP, second disease progression.

^a^ Estimated by the survival fitting implemented in R software.

^b^ Estimated in Table S3.

^c^ Estimated in Table S4.

## 3 RESULTS

### 3.1 Incremental cost‐effectiveness ratios

For Chinese patients with driver‐negative advanced or metastatic sqNSCLC, the use of sintilimab in the first‐line setting yielded 1.37 QALYs with a total healthcare cost of $12,203; reserving sintilimab treatment until the second‐line yielded 1.52 QALYs with a total healthcare cost of $14,045. Therefore, the second‐line sintilimab was associated with a greater effectiveness and a higher healthcare cost, resulting in an ICER of $12,693/QALY (Table 2).

**TABLE 2:** Summary of simulation results

Outputs	First‐line sintilimab	Second‐line sintilimab	Incremental
QALYs			
PFS health state	0.61	0.38	−0.23
FDP health state	0.49	1.01	0.52
SDP health state	0.10	0.05	−0.05
End‐stage disease health state	0.17	0.08	−0.09
Death health state	0.00	0.00	0.00
Overall QALYs	1.37	1.52	0.15
Costs, $			
PFS, $	6515	4921	−1594
FDP, $	1237	6365	5128
SDP, $	1678	809	−869
End‐stage disease, $	1569	760	−809
Death, $	1204	1190	−14
Overall costs, $	12,203	14,045	1842
ICER, $/QALY			12,694

Abbreviations: FDP, first disease progression; ICER, incremental cost‐effectiveness ratio; QALY, quality‐adjusted life‐years; SDP, second disease progression.

### 3.2 Sensitivity analysis

**FIGURE 2:**
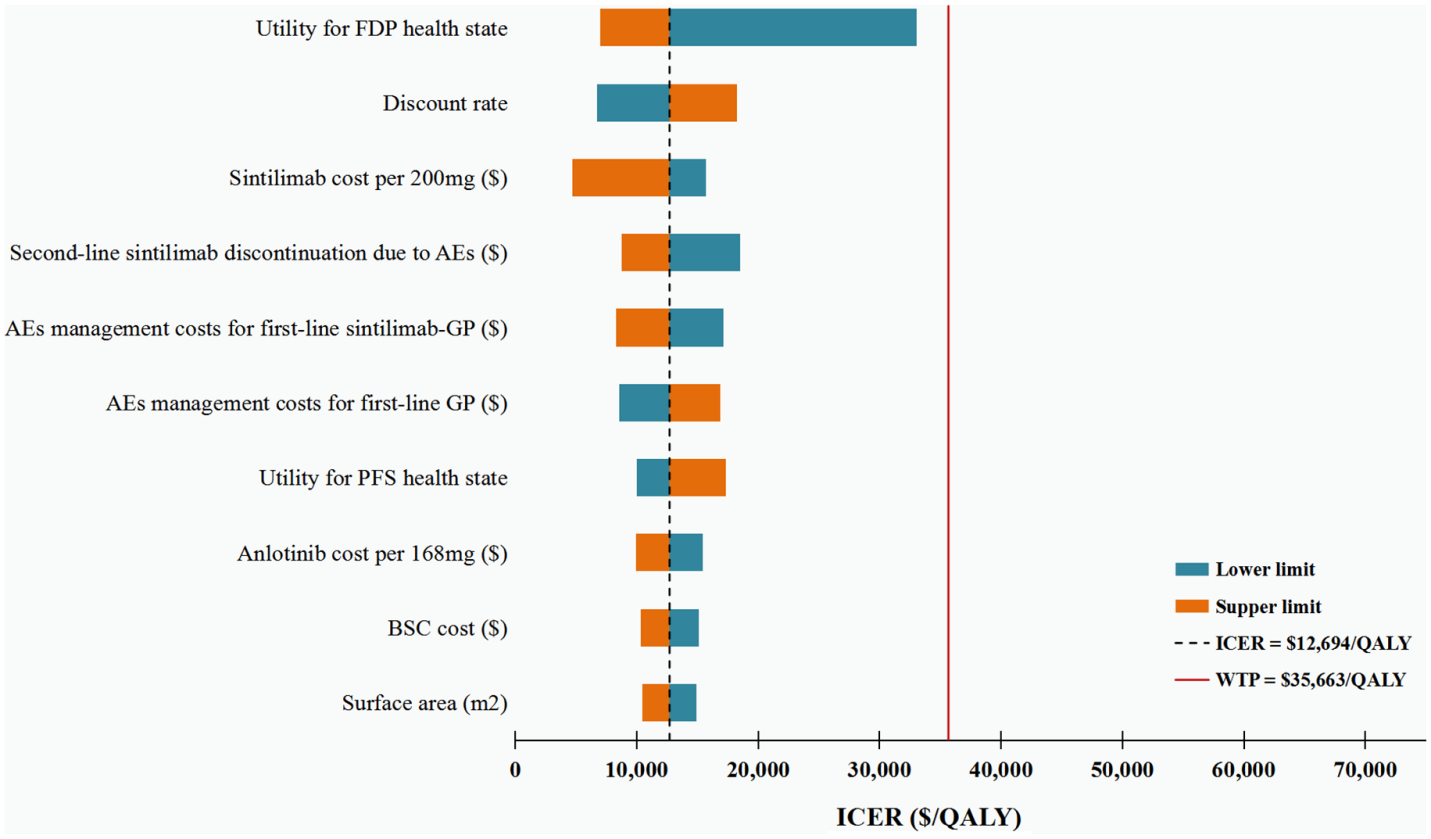
Result of deterministic sensitivity analysis. The top 10 parameters most relevant to ICERs are displayed. AEs, adverse events; BSC, best supportive care; FDP, first disease progression; GP, gemcitabine plus platinum; ICER, incremental cost‐effectiveness ratios; PFS, progression‐free survival; QALY, quality‐adjusted life‐years; WTP, willingness‐to‐pay.

The results of DSA are illustrated by the tornado diagram (Figure 2). It suggested that, variations in any input parameter within a wide and plausible range did not result in the ICERs surpassing the WTP threshold of $35,663/QALY. This means that our main finding were robust.

The results of PSA are illustrated by the cost‐effectiveness acceptability curve (Figure S4). Compared with first‐line use of sintilimab, second‐line use of sintilimab was cost‐effective in 67.6% of the 1000 iterations, when assuming a WTP threshold of $35,663/QALY (Figure S4).

## 4 DISCUSSION

In 2015, China spent more than $30 billion USD on medical expenditure for cancer treatment, which brought a significant economic burden to the country.^26^ In view of this and also the fact that the new NSCLC cases continue to rise rapidly, more studies are warranted to provide useful evidence to inform a reasonable health resource allocation.^27^ Though the immunotherapy has been known as an effective treatment for cancers, their pricy costs has made their unaffordable for most of patients and thus the cost‐effectiveness of their wide use remains to be determined.^28^ Immunotherapy is a promising therapeutic option that can be used as either a first‐line or second‐line treatment among patients with driver‐negative advanced or metastatic sqNSCLC, but a reuse in second‐line after a first‐line treatment is generally not recommended.^13^ Therefore, oncologists face challenges in making right decisions about developing proper treatment strategies, underscoring the need for an economic evaluation.

This study uniquely demonstrated the cost‐effectiveness superiority of using sintilimab in a second‐line treatment over in the first‐line. This finding highlights the importance of considering the commencing time of using immunotherapy in daily clinical practice that could make a substantial difference in its cost‐effectiveness. As an overtreatment with immunotherapy may be counterproductive,^29^ a question regarding whether an immunotherapy is better to be used in the first‐line setting or reserved until second‐line needs to be addressed. Only one published pharmacoeconomic study has attempted to address this question.^30^ However, they concluded that the first‐line use of an imported anti‐PD‐1/L1 drug (pembrolizumab) dominated its reserved use until second‐line, which is contrary to our conclusion. The main reason for this inconsistency may due to the superior clinical benefit associated with first‐line use of pembrolizumab; however, the survival benefit was not evident for first‐line sintilimab when compared with the reservation of its use until the second‐line treatment. Future investigations are needed to provide more robust evidence on this.

In our study, we have adequately considered the role of treatment‐related AEs in a cost‐effectiveness evaluation. To do this, we made a series of model assumptions to reflect the real‐world situations. First, in terms of effectiveness estimation, we incorporated three temporary health states called “discontinued therapy” into the model and estimated the corresponding transition probabilities based on AE‐related data reported in the relevant clinical trials. Secondly, we considered the AEs costs in the model which were derived from local comprehensive hospitals. Sensitivity analysis found that although we fluctuated these AEs‐related parameters to a wide extent, our results remained robust.

Improving the accessibility and affordability of anticancer drugs has consistently been the Chinese government's top priority. China has implemented several major health‐care reform policies which included funding domestic scientists to develop anticancer drugs,^31^ running price reduction negotiations with pharmaceutical companies,^32^ and expanding the National Reimbursement Drug List to incorporate anticancer drugs.^33^ Against this background, a number of domestic anticancer drugs with favorable efficacy while an affordable price have been made available on market now. Sintilimab is an anti‐PD‐1 drug similar to the imported drug pembrolizumab, but it is more likely to be widely used in clinical practice because its overwhelming price advantage compared with pembrolizumab ($ 334 vs. 5554 per 3‐week dosage). This underpins the necessity of assessing the cost‐effectiveness of domestic anticancer drugs.^19^

This study, to our knowledge, is the first one to evaluate the cost‐effectiveness of using immunotherapy in the first‐line versus reserving it until the second‐line for advanced or metastatic NSCLC. In addition, this study is also the first to investigate the cost‐effectiveness of domestic anti‐PD‐1 antibody sintilimab among Chinese patients with driver‐negative advanced or metastatic sqNSCLC. Our DSA results demonstrated our conclusion is robust despite the great fluctuation of the cost per cycle of sintilimab. Under the control of the Chinese government on the price of domestic anti‐cancer drugs, drugs in the same class usually have a similar price.^31^, ^32^, ^33^ Moreover, a recent real‐world study reported no statistically significant differences in the efficacy and safety between different PD‐1/L1 inhibitors for Chinese NSCLC patients.^34^ Therefore, we could conservatively conclude that for Chinese patients with driver‐negative advanced or metastatic sqNSCLC, reserving the use of domestic PD‐1/L1 inhibitors until second‐line represents a cost‐effective treatment strategy compared with using it in the first‐line.

This study has several notable strengths. First, we synthesized all available clinical trials data through economic modeling to enable a cost‐effectiveness comparison of first‐line use of sintilimab with reserving it until second‐line among Chinese patients with driver‐negative advanced or metastatic sqNSCLC. Second, we incorporated a real‐world therapeutic paradigm in the model, such as the use of second‐line sintilimab for patients with front‐line chemotherapy failure, the use of second‐line docetaxel in individuals in whom the front‐line immunotherapy had failed. And the third‐or further‐line use of anlotinib for patients who progressed after the second‐line treatment. Third, treatment discontinuation caused by unacceptable toxicity before a progression was considered in our model to reflect clinical practice.

This study also had some limitations. First, although this economic evaluation was based on 3 randomized clinical trials with a same patient group, the potential heterogeneity across these trials cannot be ruled out in the absence of individual patient data. Second, owing to dearth of quality‐of‐life data for sintilimab, we used Chinese NSCLC patient‐specific health utilities reported in the previous literature to inform the model; sensitivity analysis showed that the uncertainty in health utilities did not substantially change our results. Third, the post‐trial clinical outcomes for patients with driver‐negative advanced or metastatic sqNSCLC remain uncertain, although the long‐term survival was inferred from KM curves using validated extrapolation techniques. Fourth, for parameters whose corresponding 95% CIs were not available, a uniform variance of baseline value plus or minus 50% was assumed in our sensitivity analysis; and our model was shown to be not particularly sensitive to these parameters. Fifth, considering the unique perspective from the Chinese healthcare system, our results may not be generalized to other healthcare systems.

## 5 CONCLUSIONS

For Chinese patients with driver‐negative advanced or metastatic sqNSCLC, reserving the use of domestic PD‐1/L1 inhibitors sintilimab until second‐line treatment represents a cost‐effective treatment strategy compared with its use in the first‐line. This study, to the best of our knowledge, is the first one that evaluated the cost‐effectiveness of using an immunotherapy in first‐line treatment versus reserving it until second‐line for advanced or metastatic NSCLC. Our findings are useful to inform Chinese healthcare policymakers regarding the optimized treatment strategies of use of domestic PD‐1/L1 inhibitors sintilimab.

## AUTHOR CONTRIBUTIONS

**Rihua Cheng:** Data curation (equal); formal analysis (equal); investigation (equal); resources (equal); software (equal); validation (equal); visualization (equal); writing – original draft (lead). **Zhen Zhou:** Data curation (equal); formal analysis (equal); investigation (equal); resources (equal); software (equal); validation (equal); visualization (equal); writing – review and editing (lead). **Qiao Liu:** Conceptualization (lead); formal analysis (equal); methodology (lead); project administration (lead); software (equal); supervision (lead); validation (equal).

## CONFLICT OF INTEREST

Rihua Cheng, Zhen Zhou and Qiao Liu declare that they have no conflict of interest.

## ETHICS STATEMENT

The study was exempt from gaining individual consent, and no ethical approval was required for the study.

## Supporting information


Appendix S1.

## Data Availability

The original contributions presented in the study are included in the article/Supplementary Material; further inquiries can be directed to the corresponding author.
