# Supplementary material for: Cost‐effectiveness of first‐line versus second‐line use of domestic anti‐PD‐1 antibody sintilimab in Chinese patients with advanced or metastatic squamous non‐small cell lung cancer
Source: Cancer Med. 2022 Nov 13;12(6):7389–97. doi: 10.1002/cam4.5440 (PMC10067111; doi:10.1002/cam4.5440)
Supplement: Supplementary file 1 — Appendix S1. [file CAM4-12-7389-s001.docx]

**Additional file**

Table S1. Treatment dosage and administration schedule for each line treatment.

Table S2. AIC and BIC statistics for alternate parametric survival distributions.

Table S3. Proportion and probability of second- or third-line treatment discontinuation due to AEs

Table S4. Proportions, costs and disutilitis of grade III/IV AEs considered in the model.

Figure S1. Model Validation for first-line treatments used in the model. OS, overall survival; PFS, progression-free survival; KM, Kaplan-Meier.

Figure S2. Model Validation for second-line treatments used in the model. OS, overall survival; PFS, progression-free survival; KM, Kaplan-Meier.

Figure S3. Model Validation for third-or further-line treatment used in the model. OS, overall survival; PFS, progression-free survival; KM, Kaplan-Meier.

Figure S4. Cost-effectiveness acceptability curves. QALY, quality-adjusted life-year.

Table S1. Treatment dosage and administration schedule for each line treatment.

| **Regimens** | **Treatment dosage** | **Administration schedule** |
| --- | --- | --- |
| First-line sintilimab -GP | Sintilimab, 200 mg fixed | On day 1, consecutive cycles |
|  | Gemcitabine,1.0 g/m^2^ | On day 1 and day 8, a maximum of 6 consecutive cycles |
|  | Cisplatin,75 mg/m^2^ (38.5%)  or carboplatin, AUC 5.0 mg/ml/min (61.5%) | On day 1, a maximum of 6 consecutive cycles |
| First-line GP | Gemcitabine,1.0 g/m^2^ | On day 1 and day 8, a maximum of 6 consecutive cycles |
|  | Cisplatin,75 mg/m^2^ (37.1%)  or carboplatin, AUC 5.0 mg/ml/min (62.9%) | On day 1,a maximum of 6 consecutive cycles |
| Second-line sintilimab | Sintilimab, 200 mg fixed | On day 1, consecutive cycles |
| Second-line docetaxel | Docetaxel,75 mg/m^2^ | On day 1, a maximum of 6 consecutive cycles |
| Third- or further-line anlotinib | Anlotinib,12 mg/d | Daily for the first 14 days of consecutive cycles |

Abbreviations: GP, gemcitabine plus platinum; AUC, Area Under Curve.

Table S2. AIC and BIC statistics for alternate parametric survival distributions.

| **Parametric Distribution** | **First-line sintilimab-GP** | | | | **First-line GP** | | | | **Second-line sintilimab** | | | | **Second-line docetaxel** | | | | **Third-or further-line anlotinib** | | | |
| --- | --- | --- | --- | --- | --- | --- | --- | --- | --- | --- | --- | --- | --- | --- | --- | --- | --- | --- | --- | --- |
|  | **OS data** | | **PFS data** | | **OS data** | | **PFS data** | | **OS data** | | **PFS data** | | **OS data** | | **PFS data** | | **OS data** | | **PFS data** | |
|  | **AIC** | **BIC** | **AIC** | **BIC** | **AIC** | **BIC** | **AIC** | **BIC** | **AIC** | **BIC** | **AIC** | **BIC** | **AIC** | **BIC** | **AIC** | **BIC** | **AIC** | **BIC** | **AIC** | **BIC** |
| Exponential | -192 | -188 | -79 | -76 | -259 | -255 | -60 | -56 | -325 | -320 | -161 | -156 | -267 | -263 | -140 | -137 | -183 | -179 | -205 | -200 |
| Weibull | -265 | -260 | -117 | -112 | -294 | -288 | -166 | -160 | -382 | -375 | -200 | -194 | -266 | -259 | -157 | -152 | **-192** | **-196** | **-276** | **-280** |
| Log-normal | -287 | -282 | -142 | -137 | -320 | -314 | -150 | -144 | -359 | -352 | -248 | -241 | -328 | -321 | -159 | -154 | -188 | -183 | -210 | -204 |
| Log-logistic | **-294** | **-298** | **-185** | **-189** | **-328** | **-322** | **-175** | **-169** | **-386** | **-389** | **-252** | **-246** | **-334** | **-328** | **-162** | **-166** | -184 | -178 | -218 | -212 |
| Gompertz | -284 | -276 | -175 | -168 | -309 | -301 | -159 | -151 | -365 | -356 | -238 | -229 | -232 | -223 | -142 | -135 | -160 | -152 | -270 | -261 |

Abbreviations: GP, gemcitabine plus platinum; OS, overall survival; PFS, progression-free survival; AIC, Akaike information criterion; BIC, Bayesian information criterion.

Table S3. Proportion and probability of treatment discontinuation due to AEs.

| **Regimen** | **Number of as-treated patients** | **Number of patients experiencing specific events** | **Median OS** | **Proportion** | **Instantaneous rate** | **1-cyle probabilities** |
| --- | --- | --- | --- | --- | --- | --- |
| First-line sintilimab-GP | 179 | 18 | 20.01^a^ | 0.100559 | 0.003707 | 0.003701 |
| First-line GP | 178 | 15 | 17.72^a^ | 0.084270 | 0.003478 | 0.003472 |
| Second-line sintilimab | 144 | 18 | 11.79 | 0.125000 | 0.007928 | 0.007897 |
| Second-line docetaxel | 130 | 7 | 8.25 | 0.053846 | 0.004696 | 0.004685 |
| Third- or further-line anlotinib | 296 | 31 | 10.70 | 0.104730 | 0.007237 | 0.007211 |

Abbreviations: AEs, adverse events; GP, gemcitabine plus platinum; OS, overall survival;

^a^The median OS for first-line sintilimab-GP and GP were estimated from the best-fit parametric model, because these is no relevant data provided in the latest published ORIENT-12 research report.

The following formula was applied to convert the proportion of patient experiencing AEs-related treatment discontinuation event in clinical trial period into a 1-cylce probability: $P=1-exp(-rt)$, where p indicates the probability, r is the instantaneous rate and t is the time period.

Table S4. Proportions, costs and disutilities of grade III/IV AEs considered in the model.

| AEs | Proportion (%) | | Cost per event ($)^a^ | Disutility |
| --- | --- | --- | --- | --- |
|  | First-line sintilimab-GP | First-line GP |  |  |
| Anemia | 33.5% | 32.0% | 2150.12 | /^c^ |
| White blood cell count decreased | 36.3% | 36.5% | 1267.73 | /^c^ |
| Neutrophil count decreased | 48.6% | 47.8% | 1094.28 | 0.20 |
| Platelet count decreased | 45.3% | 42.7% | 1415.63 | /^c^ |
| Nausea | 1.1% | 0.6% | 16.96 | 0.12 |
| Asthenia | 1.7% | 1.1% | /^b^ | 0.07 |
| Decreased appetite | 0.6% | 1.1% | /^b^ | /^c^ |
| Pyrexia | 1.1% | 0.0% | 1423.69 | /^c^ |
| Aspartate aminotransferase increased | 0.6% | 0.0% | 292.59 | /^c^ |
| Alanine aminotransferase increased | 0.6% | 0.0% | 292.59 | /^c^ |
| Infectious pneumonitis | 14.0% | 9.6% | 1229.23 | /^c^ |
| Weight decreased | 0.0% | 0.6% | /^b^ | /^c^ |
| Hyponatremia | 6.1% | 5.1% | 316.83 | /^c^ |
| Estimated AEs Costs and disutility | | |  |  |
| AEs cost for first-line sintilimab-GP, $ | | | 2564.35 |  |
| AEs cost for first-line GP, $ | | | 2412.56 |  |
| AEs disutility for first-line sintilimab-GP | | |  | 0.100 |
| AEs disutility for first-line GP | | |  | 0.097 |

Abbreviations: AEs, adverse events; GP, gemcitabine plus platinum.

^a^These AEs management costs used in the model were investigated from local comprehensive hospitals.

^b^Based on the local oncologists’ opinions, these AEs do not require additional treatments.

^c^Disutilities regarding these AEs were not reported.

Figure S1. Model validation for first-line treatments used in the model*.*

*
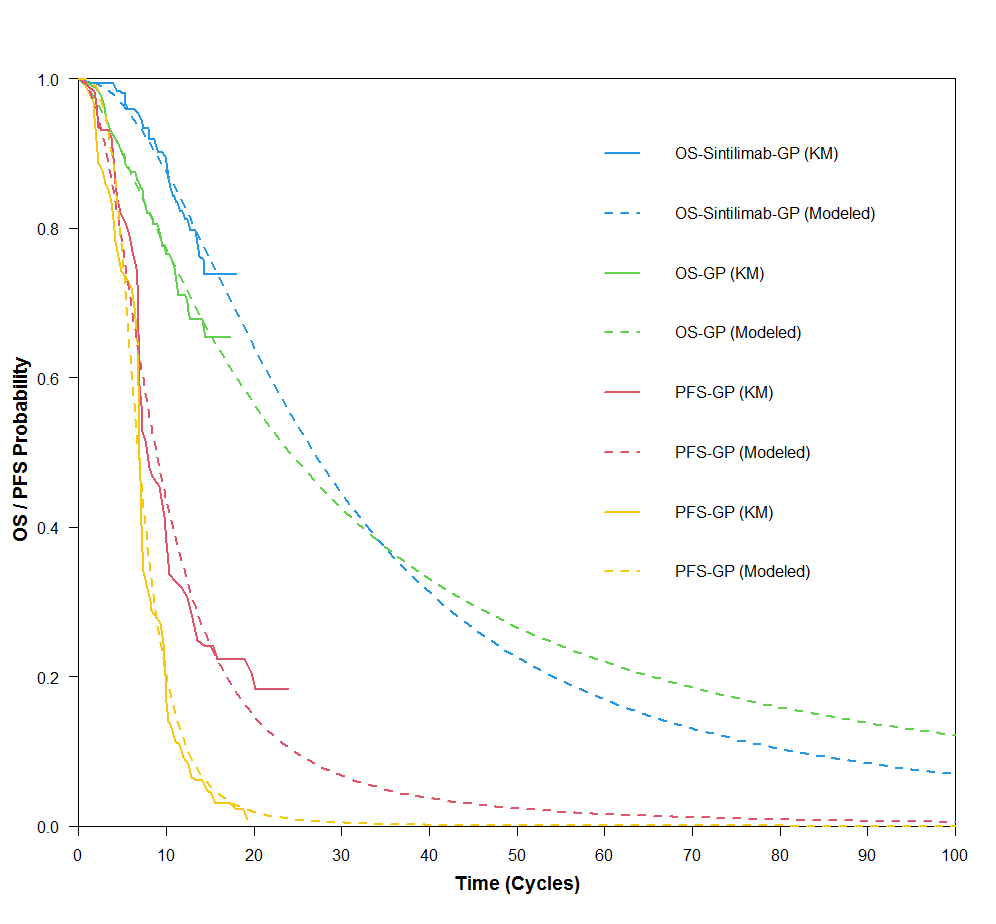
*OS, overall survival; PFS, progression-free survival; GP, gemcitabine plus platinum; KM, Kaplan-Meier.

Figure S2. Model validation for second-line treatments used in the model.


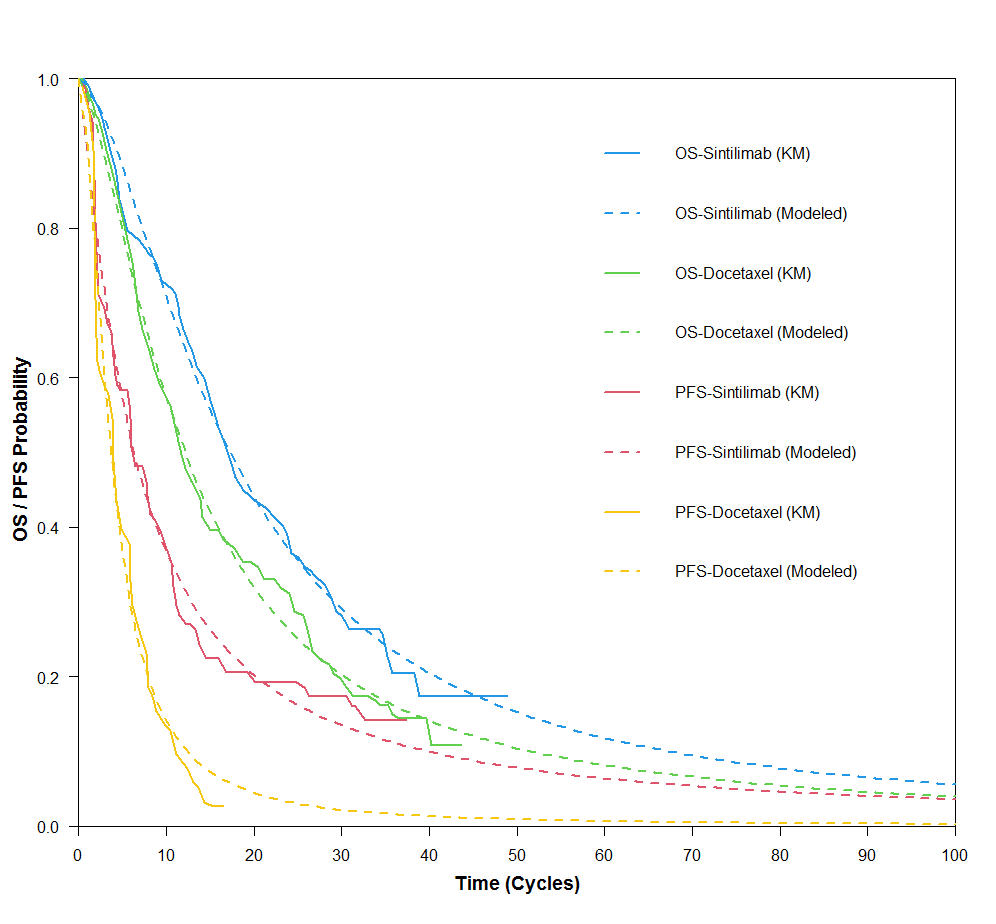
OS, overall survival; PFS, progression-free survival; KM, Kaplan-Meier.

Figure S3. Model validation for third- or further-line treatment used in the model.


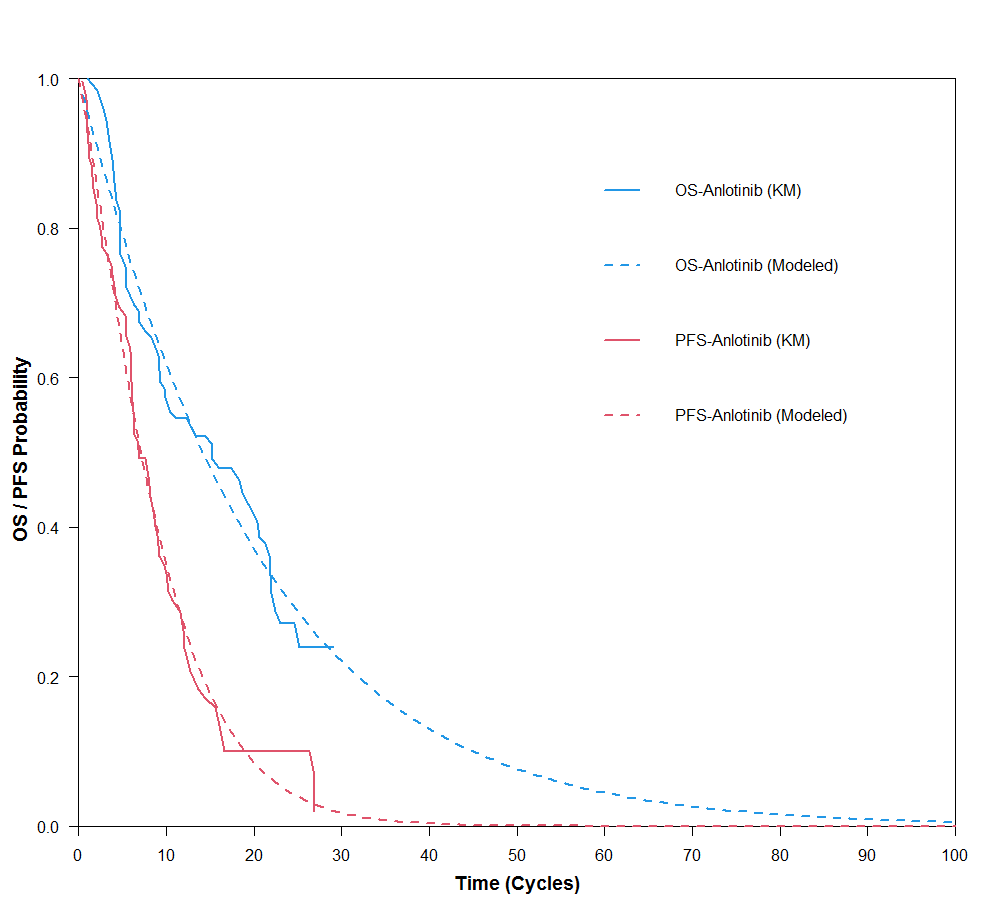
*OS, overall survival; PFS, progression-free survival; KM, Kaplan-Meier.*

Figure S4. Cost-effectiveness acceptability curves.


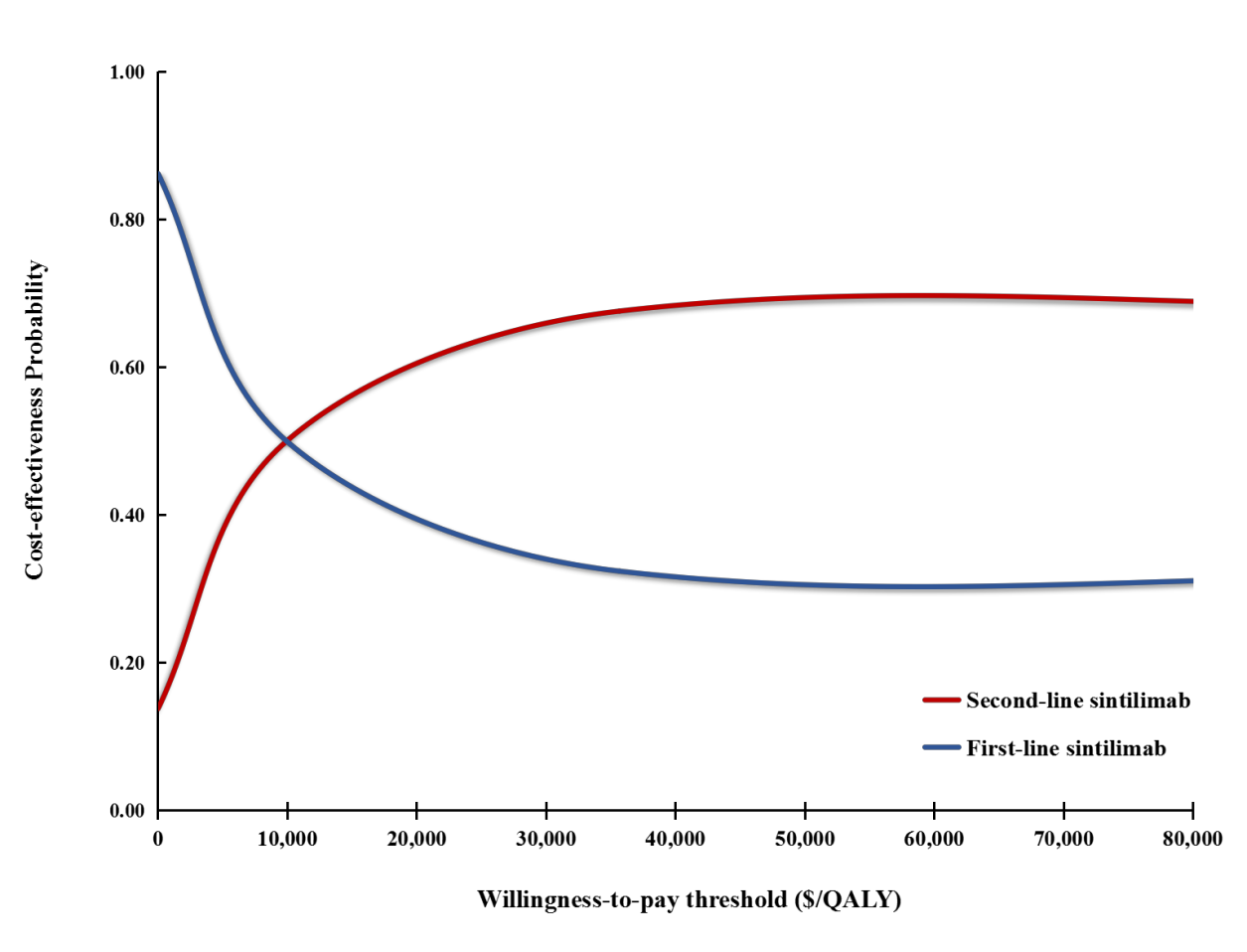
*QALY, quality-adjusted life-years.*
